# Creative Self-Efficacy and Social Skills in a Portuguese Sample of University Students: Links with Self-Esteem, Academic Achievement and Life Satisfaction

**DOI:** 10.3390/ejihpe14120195

**Published:** 2024-11-28

**Authors:** Alba González Moreno, Celeste Simões, Anabela Caetano Santos, María del Mar Molero Jurado

**Affiliations:** 1 Department of Psychology, University of Almería, 04120 Almería, Spain; mmj130@ual.es; 2 Faculty of Human Kinetics, University of Lisbon, 1495-751 Lisbon, Portugal; csimoes@sapo.pt; 3 School of Social Sciences and Humanities, ISCTE—University Institute of Lisbon, 1649-026 Lisbon, Portugal; anabela.caetano.s@gmail.com

**Keywords:** creative self-efficacy, social skills, self-esteem, academic performance, personal well-being, university students

## Abstract

Creative self-efficacy and social skills are two elements that can significantly enhance personal and professional development. The main objective of this research is to analyze the relations established between creative self-efficacy and social skills with other variables such as self-esteem, academic performance, and life satisfaction. The participants included in the methodology of this study are a total of 238 Portuguese university students. The instruments used were the Creative Self-Efficacy Scale, the Social Skills Questionnaire (CHASO), the Rosenberg Self-Esteem Scale (RSES), the Satisfaction with Life Scale (SWLS), and a series of ad hoc questions to assess academic performance. The results obtained indicate the existence of significant relations between creative self-efficacy and social skills, with the latter also being positively associated with self-esteem and life satisfaction. Analyses indicate that there are significant differences according to gender, academic performance, and the level of self-esteem of the participants. In addition, variables such as self-esteem, academic performance, and fluency act as predictors of life satisfaction. The importance of further exploring and understanding the complex relationship between creative self-efficacy, social skills, and individual well-being in the university context is discussed.

## 1. Introduction

Creativity reflects the individual’s belief in his or her ability to produce original ideas and solve problems in innovative ways [1]. In relation to this construct, the notion of creative self-efficacy encompasses each individual’s belief in his or her ability to generate original and useful ideas, as well as to produce creative solutions [2]. There has been a growing interest in exploring creative self-efficacy, a concept that has captured the attention of researchers in recent times [3]. This interest has been particularly evident in the context of the COVID-19 pandemic [4], where the importance of understanding how creative self-efficacy can influence individual and collective development and adaptation has been recognized. It has been established that creative self-efficacy is a type of creative self-belief, which stems from a person’s own confidence and belief in his or her own creative ability, and is a key factor affecting an individual’s creative output [5,6]. A recent meta-analysis indicated that creative behavior depended to some extent on individuals’ self-confidence in their creative potential [7]. This line of research seeks to unravel how confidence in one’s ability to be creative can impact diverse areas of life, from the personal sphere to the educational and work environment [8].

Creative self-efficacy influences people’s willingness to face creative challenges, as well as the effort and persistence they put into solving them [9]. Thus, people with high levels of creative self-efficacy tend to be more motivated and willing to mobilize mental resources and actions to meet the demands of the environment [10]. However, those with low creative self-efficacy may feel more likely to abandon challenging situations [11]. Regarding gender differences, a study observed that male students showed higher levels of creative self-efficacy compared to female students [12]. The idea that male students have higher creative self-efficacy has been validated in other studies [13]. Creative self-efficacy not only drives participation in creative activities, but also influences the individual’s self-esteem, acting as a precursor to greater confidence in one’s abilities [14]. Although the number of studies that have directly analyzed the relationship between creative self-efficacy and self-esteem is limited, a meta-analysis pointed out that there was a positive correlation between self-esteem and creativity, and especially a significant positive correlation between self-esteem and creative personality [15]. Focusing also on creativity, it has been found that there is a positive correlation between the level of self-esteem and creativity in young people, although no evidence was found in terms of differences according to sex [16]. Both creative self-efficacy and self-esteem are estimated to be variables that predict the level of creativity of college students [17]. Positive self-esteem, in turn, strengthens creative self-efficacy, because it impacts autonomous decision making, intrinsic motivation, emotional self-regulation, and the individual’s general well-being [18,19]. Therefore, in reference to other studies, it was established that self-esteem can act as a predictor of creative self-efficacy [20].

Research has suggested that creativity and critical thinking, components of creative self-efficacy, are positively associated with academic performance, as they influence motivation, persistence, and quality of academic work [21]. Students with high creative self-efficacy tend to engage more in challenging academic tasks and seek innovative solutions to problems, which can translate into superior academic performance [22]. A study focused on the implementation of a program based on the development of creative thinking in young people revealed that the test group obtained an improvement in their creative self-efficacy and academic performance compared to the control group [23]. Students who exhibit independence, expressiveness, and high academic aspirations tend to excel in their grades, as well as being more likely to exhibit creativity [24]. This confidence in one’s own creative abilities has been shown to have a significant influence on life satisfaction. Studies have found that people with higher levels of creative self-efficacy tend to experience greater life satisfaction [25,26].

Research such as literature reviews assessing the relationship between creative self-efficacy and social skills are scarce. However, the relationship between creativity and social skills is of particular interest in the context of personal development and social adaptation [27]. Previous research indicates that social skills can influence people’s creativity, because there are significantly direct relations between social skills and creativity [28].

A positive association is usually found between social skills and self-esteem, due to the fact that young people with higher levels of social skills have a higher level of self-esteem [29]. Research has shown that young people with some type of disorder, such as autism, who have better abilities when it comes to interacting with their peers or adults, tend to have higher self-esteem, while those with difficulties in social interaction may experience more frustration and, therefore, have lower self-esteem [30]. People with higher self-esteem show a greater willingness to communicate, share opinions, emotions or ideas, and actively participate in interactions with others in social settings [31]. On the other hand, people who experience lower levels of self-esteem tend to possess lower conflict resolution resources, and a greater tendency to be victims of violence [29,32]. Social skills such as effective communication, teamwork, conflict resolution, and collaboration are fundamental to students’ academic performance [33], as those with these skills tend to perform better in their studies [34]. The study by Huang and Zeng [35] shows that students with better academic performance also exhibited stronger socioemotional skills, with these social skills being one of the variables that have the greatest effect in explaining academic performance. Several studies underscore that these types of skills have a number of positive outcomes on the academic performance, school engagement, social relationships, and well-being of young people [36,37]. The previous literature indicates that there are gender differences in the level of students’ social skills. Although there is no consensus on this idea, studies estimated that women present reportedly higher scores in social skills compared to men [38,39].

All these benefits provided by social skills also have an impact on individuals’ life satisfaction. Related to social skills, variables such as leadership, communication, and decision making are established as predictors of self-esteem in young people, since these factors strengthen confidence in one’s own abilities by directly influencing group dynamics and success [40]. It has been indicated that several psychological aspects such as emotional regulation, social awareness, mood, and social skills are significantly correlated with life satisfaction and the social and emotional well-being of college students [41].

### Aim of the Study and Hypotheses

The aim of this research is to analyze the relation between creative self-efficacy and social skills with other variables such as self-esteem, academic performance, and life satisfaction. The initial hypotheses considered are the following:

**H1:**  *Creative self-efficacy and social skills are positively related to each other, as well as to self-esteem and life satisfaction.*

**H2:**  *Male students and young people with favorable academic progress show greater creative self-efficacy, social skills, self-esteem, and satisfaction with life.*

**H3:**  *The group of young people with higher self-esteem will show higher levels in creative self-efficacy, social skills, and life satisfaction.*

**H4:**  *Social skills, self-esteem, creative self-efficacy, and academic performance are predictors of life satisfaction.*

**H5:**  *There are differences in terms of the predictor variables of life satisfaction according to the variable repeat course.*

## 2. Materials and Methods

### 2.1. Study Design and Participants

This descriptive analysis followed the guidelines established by the STROBE statement for conducting cross-sectional studies, thus ensuring the reliability of the research [42]. This methodology offers a comprehensive view of the characteristics observed at a specific point in time, being especially useful for examining the relationship between variables at a given time.

The sample included a total of 238 university students (*M* = 20.58; *SD* = 6.86), corresponding to the mean and standard deviation of the participants’ ages, who were pursuing a degree at one of the universities in the Lisbon metropolitan area (Portugal). In relation to the distribution by gender, it is important to note the presence of self-identified women in the study, constituting 82%, while the remaining 18% were self-identified men.

The national profile of the sample indicated that most of the participants were of Portuguese nationality. However, participants of other nationalities, such as Brazilian, Dominican, or dual nationality (French–Portuguese) were also identified.

### 2.2. Instruments

The research protocol included the following instruments:

- Creative self-efficacy: Creative self-efficacy was measured using the Creative Self-Efficacy Scale [43]. This scale assesses creative self-efficacy through three factors: fluency (ability to generate a large number of ideas or solutions to a problem or task), elaboration (ability to develop and expand an initial idea, detailing and shaping it), and personality (personal characteristics that favor creativity, such as openness to new experiences or curiosity). It consists of nine items that are answered on a five-point Likert scale. The instrument showed a level of reliability using Cronbach’s alpha for each dimension of fluency (*α* = 0.63), elaboration (*α* = 0.64), and personality (*α* = 0.55). Due to the poor reliability of the personality subdimension, it was decided to eliminate this factor from the analyses of the study.
- Social skills: the Social Skills Questionnaire (CHASO) [44] was used to assess the participants’ social skills. This questionnaire, validated for the Portuguese population, consists of 40 items distributed in 10 dimensions. In addition to the dimensions, the questionnaire also assesses the overall level of social skills. Responses were scored on a five-point Likert scale. The total score on social skills showed high reliability (*α* = 0.91).
- Self-esteem: The assessment of self-esteem was based on the Portuguese adaptation of the Rosenberg Self-Esteem Scale (RSES) [45,46]. This scale consists of 10 items designed to measure feelings toward oneself on a Likert-type scale with four response options (1 = Strongly disagree; 2 = Disagree; 3 = Agree; 4 = Strongly agree). The internal consistency of the scale was good (*α* = 0.88).
- Academic performance: The academic performance of the participants was examined through questions incorporated in the questionnaire that addressed this issue. Two questions were asked, one related to the possibility of failing subjects (e.g., Have you failed any subject in the last year?) and the other focused on whether students had repeated a grade (e.g., Have you ever repeated a grade?). Both questions were answered with a dichotomous response.
- Life satisfaction: Life satisfaction was assessed using the Portuguese adaptation of the original version of this scale [47,48]. It evaluates the general satisfaction that one has with his/her life through five items. The interpretation is made considering that the higher the score, the higher the satisfaction with life. The internal consistency of the scale was good (*α* = 0.83).

### 2.3. Research and Data Collection Procedure

Data collection was carried out in several universities in the Lisbon metropolitan area, Portugal. For this purpose, contact was established with the professors teaching in various university faculties. The professors who allowed data collection among their students were visited at the beginning of the classes. During these visits, detailed information was provided on the objectives and scope of the research, obtaining informed consent from the participants. The voluntary and anonymous nature of the study was emphasized, so that students could freely decide to participate.

Data collection was carried out through an online form containing the various items of the scales used. Despite being an online form, the participants completed the surveys in the presence of a researcher with the intention of resolving any doubts. All data collection was carried out on different days during the months of October and November 2023, as part of a short stay conducted by the lead author (AGM). To ensure compliance with ethical standards, the study was approved by the Ethics Committee of the Lisbon Academic Center of Medicine under reference 168/23. This approval ensures that all practices and procedures carried out during the study respected fundamental ethical principles, guaranteeing the integrity and well-being of the participants involved in the research.

### 2.4. Data Analysis and Exploration

Data analysis was carried out using SPSS statistical software version 28 [49], a tool recognized for its efficiency in handling and interpreting complex data.

To ensure the reliability of the instruments used, Cronbach’s alpha coefficient was utilized. The interpretation of the values was based on the guidelines established by Cronbach [50], considering values below 0.5 as unacceptable, between 0.5 and 0.6 as poor, between 0.6 and 0.7 as questionable, between 0.7 and 0.8 as acceptable, between 0.8 and 0.9 as good, and above 0.9 as excellent. This analysis ensured the validity and soundness of the instruments used to measure the variables. In order to provide additional information on the participating students, a descriptive analysis was performed, revealing data on gender, age, and nationality.

To identify possible differences according to the gender of the university students and the variables analyzed, a Student’s t-test was used. The magnitude of these differences was evaluated using Cohen’s d [51], a measure that classifies the effect size as small (0.50), medium (0.50–0.80), and large (≥0.80), thus providing a complete perspective on the practical relevance of the results obtained.

To determine the differences according to self-esteem groups (g1 = low self-esteem; g2 = medium self-esteem; and g3 = high self-esteem) in comparison with the different dimensions of a healthy lifestyle and stress, an analysis of variance (ANOVA) was performed. To determine the effect size in such an analysis, eta squared (*η²*) was used, which is usually considered to be a small effect around 0.01, while a value around 0.06 indicates a moderate effect, and a value above 0.14 is considered a large effect [52].

Finally, a stepwise linear regression analysis was conducted to identify the predictor variables of life satisfaction and those that predict repeating a course in college students.

## 3. Results

### 3.1. Initial Analysis

The results obtained after analyzing the correlations between the variables included in this study show how certain variables are related to each other (Table 1).

These analyses indicate how the two factors of creative self-efficacy (fluency and elaboration) are positively related to social skills and vice versa (*r* = 0.33, *r* = 0.26; *p* < 0.001). Regarding social skills, this construct is not only positively related to creative self-efficacy, but also to self-esteem (*r* = 0.45; *p* < 0.001) and life satisfaction (*r* = 0.29; *p* < 0.001). On the other hand, life satisfaction is related to both social skills (*r* = 0.29; *p* < 0.001) and self-esteem (*r* = 0.46; *p* < 0.001), as well as the creative self-efficacy fluency factor (*r* = 0.21; *p* < 0.001). No significant correlations were found between creative self-efficacy and self-esteem, nor were significant relationships found between elaboration and life satisfaction.

### 3.2. Gender and Academic Achievement Differences in Creative Self-Efficacy, Social Skills, Self-Esteem, and Life Satisfaction

Once the existing correlations had been presented, we wanted to investigate the existence of differences according to gender (Table 2).

The analyzed data show that boys reported statistically significant higher levels in the elaboration (*M* = 10.65; *SD* = 2.20) factor related to creative self-efficacy compared to girls (elaboration: *M* = 9.79; *SD* = 2.40) (*t* = 2.14; *p* < 0.05). These differences have also been significant in the level of social skills, with boys reporting statistically significant higher levels of social skills (*M* = 132; *SD* = 21.01) than girls (*M* = 123.77; *SD* = 21.65) (*t* = 2.30; *p* < 0.05). No differences were obtained in the variables of self-esteem or life satisfaction.

Table 3 shows the differences found according to academic performance, both in terms of failing subjects and repeating an academic year.

The results show that, with respect to failing subjects, there are no differences in any of the variables analyzed. However, differences were found with respect to repeating an academic year.

University students who have never repeated a course during their academic career show statistically significant higher levels of fluency (*M* = 7.31; *SD* = 2.29) compared to those who have repeated a course (*M* = 6.33; *SD* = 2.17) (*t* = 2.19; *p* < 0.05). The same occurs with life satisfaction, since non-repeating students indicate statistically significantly higher levels of life satisfaction (*M* = 18.15; *SD* = 4.37) than those who have repeated (*M* = 15.13; *SD* = 4.94) (*t* = 3.47; *p* < 0.001).

### 3.3. Differences Between the Different Self-Esteem Groups with Creative Self-Efficacy, Social Skills and Life Satisfaction

In view of the differences according to gender and academic performance examined above, we wanted to find out if there are also differences according to the level of self-esteem. Table 4 reveals, through an analysis of variance (ANOVA), that there are disparities according to the level of self-esteem. The group of adolescents with higher self-esteem (g3) demonstrated significantly higher scores in social skills (*F* = 26.79; *p* < 0.001; *η*^2^ = 0.18) and life satisfaction (*F* = 15.54; *p* < 0.001; *η*^2^ = 0.11). No differences were obtained according to the different self-esteem groups in relation to creative self-efficacy.

### 3.4. Predictors of Life Satisfaction and Academic Performance in a University Population

A stepwise multiple linear regression analysis has been performed to find out which of the variables analyzed in this study predict life satisfaction among university students. Table 5 shows the associations between the independent variables and the dependent variable according to the effect sizes obtained (standardized *β*) and the variance explained (R-squared). A total of three models make up this regression analysis. Model I explains 21% of the variance and consists of the dependent variable (life satisfaction) and self-esteem as an independent variable. On the other hand, Model II explains 24% of the variance and consists of life satisfaction (dependent variable) and self-esteem and repeating the course as independent variables. Finally, Model III is the one that offers the greatest explanatory capacity (*R*^2^ = 0.26), and is composed of the dependent variable (life satisfaction), self-esteem, repeating a course, and fluency. This last model indicates that both the level of self-esteem and fluency positively predict life satisfaction, while repeating a course acts as a negative element in the life satisfaction of university students. Of all these variables that act as predictors, self-esteem has the greatest weight within the model. In addition, the absence of collinearity is noted in obtaining high scores in the tolerance indicators and low scores in IVF.

After learning about the predictors of life satisfaction, we wanted to examine them in terms of academic performance. Specifically, this was carried out with course repetition, since in the previous regression model it was determined that repeating a course is a predictor of life satisfaction. Thus, the participants who have repeated a grade and those who have not repeated a grade were evaluated (Table 6).

The results of the regression analysis, focused on students who have repeated a grade, reveal a single model with an explanatory capacity of 27% (*R*^2^ = 0.27). The variable that forms part of this model is self-esteem, being the one that contributes most to explaining the dependent variable (life satisfaction). The absence of collinearity is confirmed by the high scores on tolerance and low scores on IVF.

As for university students who have not repeated a grade, two models have been developed, with Model II standing out with an explanatory capacity of 22% (*R*^2^ = 0.22). Self-esteem is the most relevant predictor in this model, although significant scores are observed for both self-esteem and fluency. The absence of collinearity is established by obtaining high scores in tolerance and low scores in IVF.

## 4. Discussion

This research has made it possible to investigate the relationship between creative self-efficacy and social skills and other individual variables such as self-esteem, academic performance, and life satisfaction.

The first hypothesis of this study states that creative self-efficacy and social skills are positively related to self-esteem and life satisfaction. This hypothesis has not been completely fulfilled, because creative self-efficacy has not been found to have significant associations with self-esteem. However, it does indicate associations (only in the fluency dimension) with life satisfaction. Regarding social skills, the hypothesis is true, since it is related to both self-esteem and life satisfaction. The previous literature does indicate association between creativity, self-esteem, and life satisfaction [14,17,25,26]. This difference may be due to the fact that creativity is a polysemous construct and, in this research, we inquired about creative self-efficacy and not about creativity in general. Regarding social skills, previous studies confirm the data obtained, due to the fact that they indicate a positive relationship between social skills, self-esteem, and life satisfaction [29,31,32,41].

The second hypothesis establishes that there are differences according to gender (higher levels in girls) and academic performance (favorable academic performance) in the variables included in the study. According to gender, the results indicate that boys manifest higher levels of social and elaboration skills (creative self-efficacy) than girls. This idea is not related to the starting hypothesis, since previous studies showed that girls had higher social skills [38,39]. As for creative self-efficacy, according to other studies, there is no objective evidence that establishes differences according to sex [16]. These differences between the results and the initial hypothesis may be due to cultural or social factors, as well as the evaluation tools used. Regarding academic performance, the results indicate that participants who claim not to have repeated a grade present greater fluency (creative self-efficacy dimension) and social skills than those who have repeated a grade at some point in their educational trajectory. This idea does link to the initial hypothesis, and is related to the fact that students with creative self-efficacy engage to a greater extent with academic tasks [22]. The same occurs with students with good social skills, since having a greater ability to communicate and work in teams is linked to having better grades [33,34,35].

The third hypothesis indicates that the students’ level of self-esteem will favor higher levels of one variable or another. The results accept this hypothesis, since the statistical data obtained show that the participants included in the higher self-esteem group have greater social skills and are more satisfied with their lives. As previously mentioned, previous studies report that having a high level of self-esteem is related to better socialization skills and greater satisfaction with life [31,41].

The fourth hypothesis of this study is linked to knowing which are the predictor variables of life satisfaction. The results of the regression analysis show that self-esteem, course repetition, and fluency are the elements that significantly predict that students have greater satisfaction with life. This idea is linked to the fact that elements such as self-esteem and creativity influence aspects such as decision making or their motivation, and all of these are related to the subjects’ well-being [18,19]. Having inquired about the variables that predict life satisfaction, the idea arose to find out which variables predict this well-being in students who have repeated a grade and those who have not repeated a grade.

Based on the previous results, the fifth study hypothesis was developed, which determines that there are differences in the predictor variables of satisfaction with life according to the criterion of repeating or not repeating a course. This hypothesis has been accepted, since there are differences between the two student profiles. Life satisfaction in repeating students is predicted by self-esteem, while in non-repeating students it is predicted by both self-esteem and fluency. Previous studies highlight that people with high creative self-efficacy are more motivated and tend to be more persistent in solving challenges [9,10]. Thus, this type of student body, showing higher creative skills, is more likely to perform better academically [24].

## 5. Conclusions

To conclude, it is necessary to point out how this research has been able to verify the relationships between creative self-efficacy and social skills with certain important variables at the individual level, such as self-esteem, academic performance, and life satisfaction. The results obtained through this study give us an idea of how positive it is to promote aspects such as creative self-efficacy and social skills in university students. Thus, one of the practical implications of this work has been to determine how certain variables such as self-esteem, academic performance, and life satisfaction interfere with the intention of proposing programs that promote aspects such as social skills and creative self-efficacy within the university context. It is suggested to implement programs with the student body that integrate personal development workshops focused on self-esteem, as well as collaborative learning activities to reinforce social interaction and creativity. In addition, fostering an environment that values the diversity of ideas and experiences can improve students’ life satisfaction and self-efficacy. Universities, by implementing these strategies, can contribute significantly to the holistic development of their students, better preparing them to meet academic and professional challenges. Considering the absence of a correlation between creative self-efficacy and self-esteem in the results, it is suggested that it is important to continue investigating the different factors that may influence this relationship, since there could be mediators or moderators not contemplated in the current analysis. Therefore, a possible improvement in the practical implications of the research could be to suggest that the proposed programs not only focus on self-esteem and social skills, but also on other more specific aspects that could strengthen creative self-efficacy, such as the promotion of resilience, intrinsic motivation, or even specific strategies to enhance creativity in the academic environment. Despite such practical implications, some of the limitations that emerged during the development of the work must be taken into account. One of the main limitations is that the level of reliability of the instrument used to measure creative self-efficacy has not been as successful as that of the other variables included. Therefore, in future research, it would be advisable to measure student creativity in another way. To conclude, it is necessary to emphasize the importance of continuing to explore and understand the complex relationship between creative self-efficacy, social skills, and individual well-being in the university context. Continued studies in this field could offer even more insights and practical recommendations for improving the educational experience and personal development of students.

## Figures and Tables

**Table 1:** Descriptors and correlation matrix between creative self-efficacy, social skills, self-esteem, and life satisfaction.

		[1]	[2]	[3]	[4]	[5]
Creative self-efficacy	[1] Fluency	-				
	[2] Elaboration	0.41 ***	-			
Social skills	[3]	0.33 ***	0.26 ***	-		
Self-esteem	[4]	0.12	0.02	0.45 ***	-	
Satisfaction with life	[5]	0.21 ***	0.08	0.29 ***	0.46 ***	-
	Mean	7.19	9.95	125.29	28.69	17.77
	*SD*	2.29	2.39	21.73	5.61	4.54
	Min.	3	3	58	13	5
	Max.	15	15	175	40	25

*** *p* < 0.001.

**Table 2:** Differences according to gender (girls *n* = 195; boys *n* = 43).

		Gender				*t*	*p*	*d*
		Boys		Girls				
		Mean	*SD*	Mean	*SD*			
Creative self-efficacy	Fluency	7.65	2.40	7.09	2.26	1.46	0.146	-
	Elaboration	10.65	2.20	9.79	2.40	2.14 *	0.033	0.36
Social skills		132.14	21.01	123.77	21.65	2.30 *	0.022	0.39
Self-esteem		29.79	6.29	28.45	5.44	1.41	0.157	-
Satisfaction with life		17.14	4.35	17.91	4.58	−1.00	0.317	-

* *p* < 0.05.

**Table 3:** Differences in repeating a course and failing subjects.

		Not Failed (*n* = 207)		Failed (*n* = 31)		*t*	*p*	*d*
		Mean	*SD*	Mean	*SD*			
Creative self-efficacy	Fluency	7.13	2.31	7.58	2.18	−1.01	0.310	-
	Elaboration	10.04	2.34	9.35	2.61	1.49	0.138	-
Social skills		125.30	21.76	125.16	21.92	0.03	0.973	-
Self-esteem		28.77	5.67	28.19	5.26	0.53	0.596	-
Satisfaction with life		17.95	4.60	16.58	4.04	1.56	0.119	-
		**Not Repeated** **(*n* = 208)**		**Repeated** **(*n* = 30)**		***t***	***p***	***d***
		**Mean**	***SD***	**Mean**	***SD***			
Creative self-efficacy	Fluency	7.31	2.29	6.33	2.17	2.19 *	0.029	0.43
	Elaboration	10.00	2.36	9.57	2.59	0.93	0.349	-
Social skills		125.29	21.81	125.23	21.56	0.01	0.989	-
Self-esteem		28.87	5.61	27.50	5.56	1.24	0.214	-
Satisfaction with life		18.15	4.37	15.13	4.94	3.47 ***	<0.001	0.68

* *p* < 0.05; *** *p* < 0.001.

**Table 4:** Differences according to the different groups of self-esteem.

Scale		Groups’ Self-Esteem	*N*	Mean	*SD*	ANOVA		Difference in Averages
						*F*	Sig.	
Creative self-efficacy	Fluency	Low (g1)	69	7.04	2.04	0.69	0.501	|g1–g2|
		Mean (g2)	62	7.02	2.28			|g2–g3| *
		High (g3)	107	7.38	2.46			|g1–g3|
	Elaboration	Low (g1)	69	9.99	2.34	1.08	0.339	|g1–g2|
		Mean (g2)	62	9.58	2.42			|g2–g3| *
		High (g3)	107	10.14	2.39			|g1–g3|
Social skills		Low (g1)	69	116.26	19.35	26.79	<0.001	|g1–g2|
		Mean (g2)	62	117.50	22.82			|g2–g3| ***
		High (g3)	107	135.62	17.89			|g1–g3| ***
Satisfaction with life		Low (g1)	69	15.48	4.73	15.54	<0.001	|g1–g2| **
		Mean (g2)	62	17.90	4.11			|g2–g3|
		High (g3)	107	19.17	4.09			|g1–g3| ***

* *p* < 0.05; ** *p* < 0.01; *** *p* < 0.001.

**Table 5:** Stepwise multiple linear regression model: predictor variables of life satisfaction (total sample; *N* = 238).

Variable	B	F	β	R^2^	T	*p*
*Satisfaction with life*						
Model 1						
*Self-esteem*	0.37	64.46	0.46	0.21	8.02	<0.001
Model 2						
*Self-esteem*	0.36	10.55	0.44	0.24	7.90	<0.001
*Repeat course*	−2.52		−0.18		−3.24	<0.001
Model 3						
*Self-esteem*	0.35	5.82	0.43	0.26	7.65	<0.001
*Repeat course*	−2.27		−0.16		−2.93	0.004
*Fluency*	0.27		0.13		2.41	0.017

**Table 6:** Stepwise multiple linear regression model of those that repeated a course (repeaters and non-repeaters).

*Repeaters*						
Variable	B	F	β	R^2^	T	*p*
*Satisfaction with life*						
Model 1						
*Self-esteem*	0.46	10.35	0.51	0.27	3.21	0.003
***Non-repeaters***						
**Variable**	**B**	**F**	**β**	**R^2^**	**T**	***p***
*Satisfaction with life*						
Model 1						
*Self-esteem*	0.34	52.02	0.44	0.20	7.21	<0.001
Model 2						
*Self-esteem*	0.33	29.15	0.42	0.22	6.89	<0.001
*Fluency*	0.27		0.14		2.28	0.023

## Data Availability

The data are available upon request to the corresponding author.
